# Clinical Effectiveness of Oncological Treatment in Metastatic Colorectal Cancer Is Independent of Comorbidities and Age

**DOI:** 10.3390/cancers13092091

**Published:** 2021-04-26

**Authors:** Dora Niedersüß-Beke, Manuel Orlinger, David Falch, Cordula Heiler, Gudrun Piringer, Josef Thaler, Wolfgang Hilbe, Andreas Petzer, Holger Rumpold

**Affiliations:** 1 Department of Internal Medicine I, Wilhelminenspital, 1160 Vienna, Austria; dora.niedersuess-beke@gesundheitsverbund.at (D.N.-B.); david.falch@gesundheitsverbund.at (D.F.); heillercordula@gmail.com (C.H.); wolfgang.hilbe@gesundheitsverbund.at (W.H.); 2 Department of Hematology and Medical Oncology, Ordensklinikum Linz, 4010 Linz, Austria; manuel.orlinger@ordensklinikum.at (M.O.); andreas.petzer@ordensklinikum.at (A.P.); 3 Department of Internal Medicine IV, Hospital Wels-Grieskirchen, 4600 Wels, Austria; Gudrun.Piringer@klinikum-wegr.at (G.P.); josef.thaler@klinikum-wegr.at (J.T.); 4 Medical Faculty, Johannes Kepler University Linz, 4020 Linz, Austria; 5 Gastrointestinal Cancer Center, Ordensklinikum Linz, 4010 Linz, Austria

**Keywords:** colorectal cancer, metastases, real-life, treatment, comorbidities, elderly

## Abstract

**Simple Summary:**

Colorectal cancer (CRC) is the third most common cancer worldwide. As with many other cancers, the risk for CRC increases with age. This is also true for comorbidities, which may hamper sufficient treatment of the cancer. Due to restrictive inclusion criteria, older patients and patients with comorbidities are underrepresented in clinical trials. Comprehensive knowledge about modern effectiveness of oncological treatments in older and/or comorbid patients is sparse. Due to the lack of clinical trials, this issue is investigated in real-life settings predominantly. In our retrospective study we show that patients benefit from oncological treatments irrespective of comorbidities, measured by the age-adjusted Charlson Comorbity (aaCCI) index, and age. Differences found in treatment outcomes are marginal and are likely due to less intense treatment of comorbid or elderly patients. Balancing risk and benefit for treatment decisions should take potential under-treatment of comorbid and older patients into account.

**Abstract:**

We aimed to investigate the effectiveness of oncological treatments in metastatic CRC related to comorbidities and age. This retrospective study included 1105 patients from three oncological centers. aaCCI and CCI was available from 577 patients. An aaCCI > 3 was of the highest predictive value compared to other aaCCI-levels, CCI or age (*p* < 0.001 for all). Treatment (best supportive care (BSC), systemic treatment only (STO) and resection of metastases (ROM)) significantly prolonged survival in patients with aaCCI > 3 (STO: HR 0.39, CI 0.29–0.51; ROM: HR 0.16, CI 0.10–0.24) and patients older than 70 years (STO: HR 0.56, CI 0.47–0.66; ROM: HR 0.23, 0.18–0.30). Median overall survival was shorter in patients with aaCCI or age > 70 years and interaction for treatment type not significant for aaCCI, but significant for age older or younger than 70 years (STO: *p* = 0.01; ROM *p* = 0.02). BSC is more often considered as optimal care for patients with an aaCCI > 3 (37.6% vs. 12.4%; *p* < 0.001) or age > 70 years (35.7% vs. 11.2%; *p* < 0.001). Older patients or patients with comorbidities benefit from cancer-specific therapy independently of their age and comorbidities.

## 1. Introduction

Colorectal cancer (CRC) is the third most common cancer worldwide [1]. The incidence of CRC increases with age, which is true for comorbidities as well [2,3]. Elderly patients and patients with a higher number of comorbidities are often under-represented in clinical cancer trials, although they represent the largest group of the real-life CRC patients’ collective [4,5,6]. With the advent of modern oncological treatments, a striking improvement of overall survival (OS) in metastatic CRC (mCRC), reaching around 30 months, has been achieved over the last centuries [7,8]. This number, however, reflects a specific patient population within clinical trials, which select patients with specific prognostically relevant markers [9,10]. In real-life this number is an overestimation and not seen [11,12,13,14]. The increased OS is reported for elderly patients as well, but with much lower evidence [12,15,16,17,18,19,20]. Finding the optimal cancer treatment in the elderly is difficult, partly due to the underrepresentation of these patients in clinical trials [21,22]. Most studies addressing this clinical outcome in a frail population use age as a predictor. Results are contradictive as several studies show the same benefit and similar toxicity from chemotherapy regardless of age [23,24,25,26]. In contrast, a large analysis from the Aide et Recherché en Cancerologie Digestive group reports that the risk of death was significantly lower in younger than in older patients, which can be interpreted to signify that older patients do not gain the same benefit of treatment as younger ones do [27].

Age alone might not be the optimal predictor of clinical outcomes. Additional considerations such as comorbidities are of importance as several trials have shown that pretreatment comorbidity status is an independent predictor of OS in several tumor entities [28,29]. For surgical interventions in mCRC, the American Society of Anesthesiologists classification has been shown to be useful for patient selection [30,31]. The more broadly applicable Charlson comorbidity index (CCI) is a reliable tool to estimate prognosis based on type and number of comorbidities. This index has been tested in several cancer entities [32,33,34]. Age has been incorporated into the CCI, resulting in a more informative age adjusted CCI (aaCCI) [35]. However, CCI or aaCCI are not widely used in clinical practice or clinical trials.

Older patients or patients with comorbidities are often undertreated in the real-world setting due to the lack of comprehensive data on treatment outcomes, the reluctance of physicians to treat and the different value older patients place on having a few months longer to live. The aim of this study was to investigate the effectiveness of modern oncological treatments (systemic treatment alone, resection of metastases with or without systemic treatment compared to patients receiving best supportive care (BSC)) using aaCCI or age as predictors of OS in a real-life patient population. Analyzing a possible interaction between treatment and aaCCI, as well as treatment and age, provides a basis for determining whether the effects of different treatment approaches are dependent on comorbidities and age.

## 2. Materials and Methods

### 2.1. Patient Population

We retrospectively identified patients diagnosed with localized or metastatic CRC of any histology treated at three different oncological centers from January 2005 to December 2020. Clinical data were obtained by searching two prospective cancer registries (Ordensklinikum Linz and Klinikum Wels-Griesskirchen) or by chart review (Wilhelminenspital Vienna). Patients were eligible for inclusion if they suffered from metastatic colorectal adenocarcinoma (synchronous or metachronous metastasized) and if their first metastasis was diagnosed before 31 December 2019. Metachronous disease was defined by an interval longer than 6 months from first diagnosis and diagnosis of the first metastase. Metastases were diagnosed by comptuertomography. Left sided colorectal cancer was defined as those originating from splenic flexure, descending and sigmoid colon, as well as the rectum. Right sided colon cancer included the coecum, ascending colon, hepatic flexure and colon transversum. End of disease was defined by either death or last follow-up of the patient. The last follow-up for the analyzed cohort was 15 January 2021. Information on CCI or aaCCI was obtained retrospectively for patients treated at two centers (Ordenksklinikum Linz, Wilhelminenspital Vienna).

### 2.2. Statistical Analysis

The endpoint of this study was OS defined as the time between first diagnosis of metastatic disease and death. Kaplan–Meier-estimates and curves were used as descriptive measures for survival data; the reverse Kaplan–Meier method was used for calculation of median follow-up time. Cox proportional hazard regression models were used to investigate the effects of different CCI or aaCCI-categories on survival. The assumptions of Cox regression were checked for with appropriate methods. Outliers and influential cases were deleted for all analyses after identification by sample size-dependent cutoff and graphical check for deviance (R function ggcoxdiagnostics) according to Patterson et al. [36]. Different Cox models were compared by chi-squared-test to find the CCI or aaCCI categorization with the strongest association. Predictors with the lowest *p*-value and the highest likelihood-ratio within the CCI and aaCCI groups were chosen for comparison by chi-squared-test to identify the model with the highest predictive power. Interaction effects of aaCCI ≤ 3 or higher and treatment groups, as well as age ≤ 70 years or above and treatment groups, were analyzed to investigate if treatment had a different effect on survival dependent on aaCCI category or age. This was done in a model including the whole dataset including all treatment groups. In addition, Cox models were performed on sub datasets by age group or aaCCI category with treatment as the predictor. A comparison of 90% confidence intervals of the hazard ratios was used for a more precise interpretation of the interaction results in these subgroups. Absolute and relative frequencies were reported for categorical variables and median and IQR for continuous variables. Cross tabulation and Fishers Exact tests were used to investigate the differences of categorical parameters between aaCCI- and CCI-categories and age groups. For all analyses, the R version 3.6.3 (R core Team, 2020) was used with the survival (survival analyses, Kaplan–Meier-analyses, cox-regression, diagnostics), survminer (survival-curves, diagnostics) and tableone (tables, frequencies). A significance level of 5% was assumed in all analyses.

## 3. Results

### 3.1. Patients

**Figure 1:**
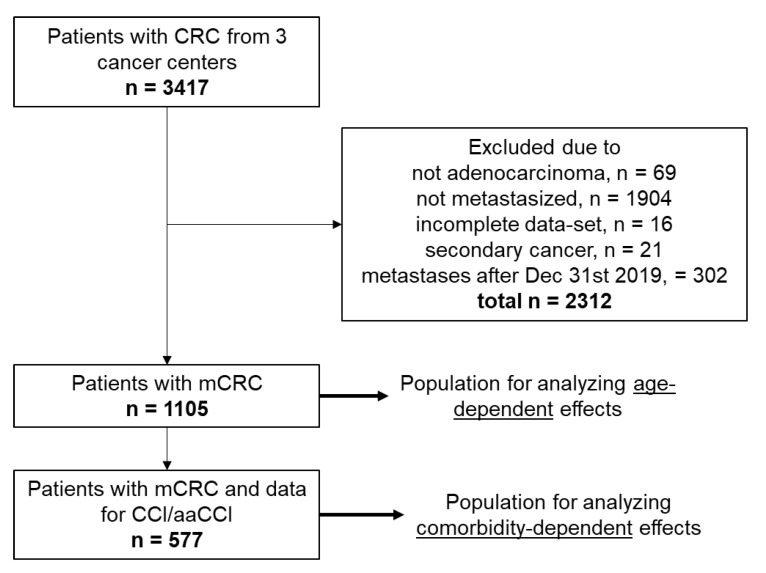
Patient selection.

A total of 3417 patients with CRC were identified in the databases of the three participating cancer centers. From these, 69 were excluded due to a histology other than adenocarcinoma; 1904 were excluded because of localized stage; 16 were excluded due to an incomplete dataset; 21 were excluded due to a second cancer, and 302 were excluded because the metastatic disease was diagnosed after 31 December 2019. This resulted in 1105 patients diagnosed with mCRC and treated at the participating centers from 1 January 2005 to 31 December 2019 and included in the analysis (Figure 1). Median age was 69 years, 37.4% were female, 62.6% were male. Most of the patients received systemic treatment alone (53.4%), followed by multimodal treatment by resection of metastases with or without systemic treatment (24.4%). A proportion of 22.2% of the patients were not treated at all. First line treatment was given to 69% of the patients, second line to 44.5%, and third line or more to 25.9%. Most of the patients had a left sided CRC (81.9%), located in the colon (64.3%). Furthermore, 69.8% of the patients suffered from synchronous disease. At diagnosis of metastatic disease, either synchronous or metachronous, 70.8% of the patients showed single organ metastasis. Mutational status of RAS was available in 79.5% of patients. For BRAF mutational status was available in only 36.7% of the patients (Table 1). From 577 patients, CCI and aaCCI were available. From these 63% had a CCI of 0, and 65.3% of the patients had an aaCCI of ≤3. Median follow-up time was 75.95 months (IQR 41.46; 107.13) for the whole population.

### 3.2. Prognostic Value of CCI and aaCCI

The highest predictive value for CCI was obtained for patients with CCI zero compared to patients with a CCI greater than zero (CCI-0; HR 1.49; CI 1.27–1.75). An aaCCI ≤ 3 or above (aaCCI-3) showed the highest predictive value when aaCCI was used as the predictor (HR 1.82; CI 1.54–2.14). The model with aaCCI-3 as the predictor showed a significantly better fit than the model with CCI-0 as the predictor (log likelihood = −2431.1 vs. −2421.7, 2(0) = 18.82, *p* < 0.001). Therefore, the model with aaCCI-3 as the predictor in terms of comorbidity was used for further analyses.

### 3.3. Treatment Outcomes According to Comorbidities and Age

**Figure 2:**
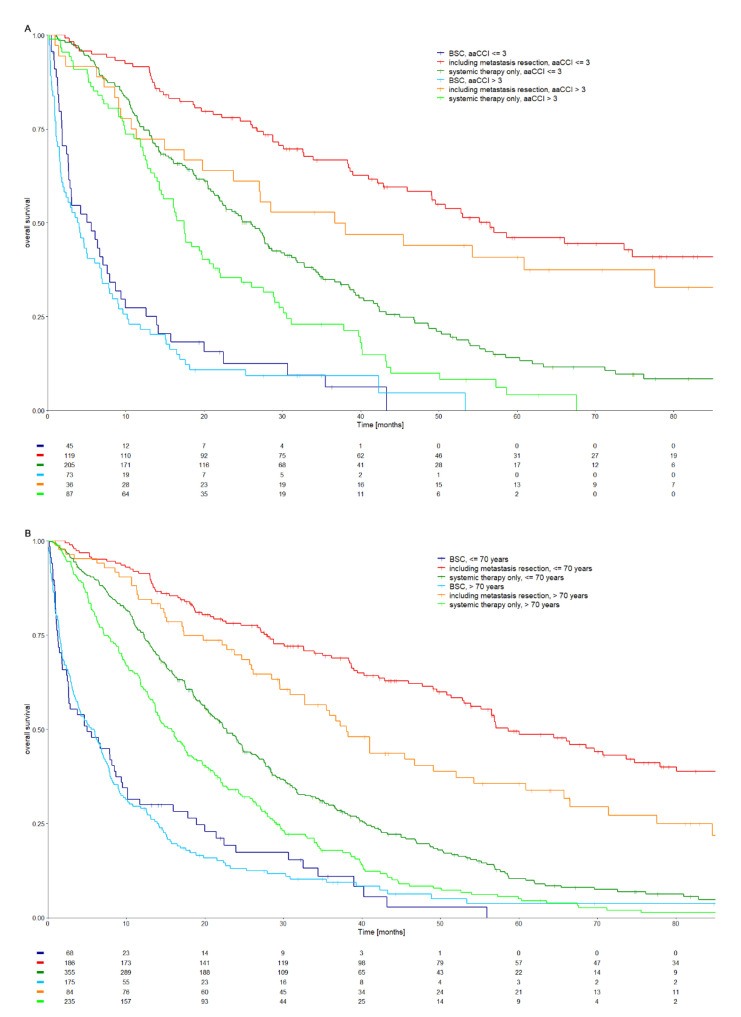
Overall survival by treatment type and age-adjusted Charlson Comorbidity Index (**A**) or age (**B**).

In the overall population, treatments including resection of metastases were the most effective therapy (HR 0.16; CI 0.13–0.20), followed by systemic treatment alone (HR 0.45; CI 0.38–0.52) compared to BSC. These effects were observed independently of comorbidities and age. Patients with an aaCCI > 3 showed a benefit from resection (HR 0.16; CI 0.10–0.24) or systemic treatment alone (HR 0.39; CI 0.29–0.51) compared to the respective BSC group. Similar treatment benefits were observed for patients older than 70 years for multimodal treatment including resection of metastasis (HR 0.23; CI 0.18–0.30) or systemic treatment only (HR 0.56; CI 0.47–0.66). These treatment benefits were more pronounced in patients with an aaCCI of ≤3 (HR for resection of metastasis 0.12; CI 0.08–0.17; HR for systemic therapy alone 0.3; CI 0.22–0.39). However, these outcome differences did not show a significant interaction between treatment group and aaCCI (*p* = 0.52 for resection of metastasis; *p* = 0.29 for systemic treatment alone). Yet, interaction did gain significance between treatment group and younger or older than 70 years of age (*p* = 0.02 for resection of metastasis; *p* = 0.01 for systemic treatment alone). Results are visualized in Figure 2A,B; HR and 90% CI’s for visualizing interaction are given in Table 2; a detailed view on the interaction analysis rendering *p*-values for interaction is given in Appendix A (Table A1).

### 3.4. Treatment Differences in Comorbid and Elderly Patients

Patients with an aaCCI > 3 were significantly more often considered for BSC (37.6% vs. 12.4%; *p* < 0.001). If treated, patients with an aaCCI > 3 were less often treated multimodally by metastasis resection and systemic treatment (9.6% vs. 24.9%; *p* > 0.001). The rate of patients in the aaCCI > 3 group receiving one treatment line only was similar to patients with an aaCCI ≤ 3 (28.9% vs. 29.2%; *p* = 1.000). However, the rate of patients receiving two lines of treatment dropped significantly in the aaCCI > 3 group (11.7% vs. 26.5%; *p* < 0.001). Consecutively, less patients with an aaCCI > 3 received three or more treatment lines (11.7 vs. 26.5%; *p* = 0.001). The reduction in treatment intensity occurred between the applications from first line to second line treatment. Out of all patients receiving first line treatment, a significantly lower proportion of patients with aaCCI > 3 received second line treatment (46.2% vs. 63.6%; *p* = 0.002). In contrast, the rate of patients receiving a third line or more of therapy was similar in both aaCCI groups (aaCCI > 3 54% vs. aaCCI ≤ 3 48.1%; *p* = 0.526). Similar results were obtained in patients younger or older than 70 years. Patients > 70 years were more often considered for BSC (35.7% vs. 11.2%; *p* < 0.001). If patients > 70 years received treatment, they were less frequently treated by multimodal treatment (9.6% vs. 24.9%; *p* < 0.001). Metastasis resection alone was comparable between the two age groups (≤70 years 9.5% vs. 7.9%; *p* = 0.339). Moreover, the rate of patients receiving at least one line of systemic treatment was comparable between the two age groups (≤70 years 23.3% vs. 26.6%; *p* = 0.208). Two treatment lines were administered significantly less often in patients >70 years (13.3% vs. 23.0%; *p* < 0.001), which continued in that there was a lower frequency of third line treatment or more in this group (16.5% vs. 33.0%; *p* < 0.001). This reduction in systemic treatment intensity again takes place between first and second line treatment. Out of all patients receiving first line treatment, a significantly lower proportion of patients received a second line therapy (52.9% vs. 70.6%; *p* < 0.001). In contrast, a similar proportion of patients received third line treatment after second line treatment was given (55.4% vs. 59.0%; *p* = 0.486). Results are summarized in Table 3.

## 4. Discussion

The main finding of our study is that older patients or patients with comorbid conditions benefit from cancer specific therapy independently of their age and comorbidities. Our data show that even in a real-life setting first-line systemic therapy, resection of the primary tumor and the elimination of metastases considerably prolong survival in these vulnerable patient populations.

Morishima and colleagues found in a large retrospective analysis of more than 2600 patients with colorectal, lung and gastric cancer that comorbidities should receive more attention for risk adjustment [37]. In line with previous data, our study confirms that apart from age, comorbidities have a high relevance as a prognostic tool in patients with metastatic CRC [28,38]. In our study aaCCI had a higher predictive value than CCI, which focuses on comorbidities not including age. Differences for aaCCI and CCI are known for several cancers, including localized CRC, but this has been not evaluated in mCRC [37,39].

However, our data also indicate that comorbid and older patients still benefit considerably from systemic and local treatments and that the putative negative effect of comorbidities on treatment toxicities needs to be weighed carefully against the expected benefit of cancer therapy. Our findings are interesting since—based on recommendations to withhold aggressive treatments in comorbid patients [28]—elderly patients with comorbidities are often treated less aggressively and thus have a worse survival than those with no concomitant diseases [33]. In contrast, our data in a real-world setting show that CRC patients should not be denied effective treatment options simply based on their comorbidities. This is even true for a more elderly patient subset.

It is difficult to prove an interaction between comorbidities or age and oncological treatment. We could not find an interaction for aaCCI and oncological treatment but did find an interaction between age (older or younger than 70 years) and oncological treatment, which, however, was barely significant (*p* = 0.02 for resection of metastasis; *p* = 0.01 for systemic treatment alone). If age is used as a metric variable, significance cannot be reached. Differences in survival between frail and not frail patients in our data set are more likely due to less intense treatment of patients with an aaCCI > 3 and patients older than 70 years. This is because older or comorbid patients were significantly less often treated by intensive therapy and received significantly less systemic treatment lines (Table 3). Furthermore, RAS analysis as a decision criterion for treatment decision was more often performed in younger and not frail patients. This was accompanied by a significantly less frequent use of monoclonal antibodies in these patient populations. Our findings are comparable to the results of the retrospective DISCO-study showing that older patients receive less aggressive treatment and, if treated, have a higher probability of receiving monotherapy instead of combination chemotherapy or additional monoclonal antibodies [40]. Interestingly, other parameters like the distribution of CCI and the frequency of KRAS/RAS analyses between the DISCO-study and our larger population are comparable, which underlines the reliability of our dataset.

The reason why intensive treatments have been withheld from multimorbid and elderly patients with metastatic CRC might be that the evidence supporting the feasibility of these therapies, including radical surgery of the primary tumor and eradication of metastases, has been limited so far [17,18,20]. In addition, findings from the adjuvant setting suggest that in the early stage of the disease, comorbidities have a higher impact on life expectancy than the tumor itself [41,42]. This might be different in the advanced setting. For example, Boakye et al., while confirming the importance of comorbidities in the adjuvant setting, recently stated that comorbidities become less important in the advanced setting with respect to overall prognosis [43]. This underlines the importance of optimally treating the leading cause of mortality, the cancer itself, even in an elderly or multimorbid population. In addition, we did not observe a difference in survival of patients receiving BSC. This speaks against a more indolent biological course of the disease in older patients, which is a commonly shared opinion among clinicians based on differences in biological features [44,45]. Thus, it is important to offer effective treatments to multimorbid and elderly patients. Focusing on the optimization of comorbid conditions is a key issue in these cases. Preliminary data show that the probability of completing a planned systemic treatment is higher if comorbidities are extensively managed and can potentially be increased by interdisciplinary care consisting of oncologists working together with geriatricians [46,47].

Age is a common criterion used for treatment decisions including intensity of treatment. In the literature, the definition of young and older patients is commonly done by using 70 years and over as a cut-off for older patients [40]. We show that predicting survival by either aaCCI or age older or younger than 70 years have almost the same predictive value in terms of median OS (Figure 1 and Table 2), which makes age an easy proxy tool to support decision making in clinical practice in terms of considering additional care for comorbid conditions. To our knowledge, such a comparison has not been reported yet.

In conclusion, our data confirm the significant role of age and comorbidities on the outcome of patients with mCRC and also show that intensive treatment approaches have a similar clinical effectiveness compared to younger or non-comorbid patients. This implies that tumor biology in terms of the natural course and sensitivity to treatment is similar in different patient groups. Modern oncology provides a wide range of treatments to tailor treatment according to patient specific conditions like comorbidities. This is, however, challenging. Yet, our study shows that treatment decisions in older patients or patients with comorbidities should be similar to that of other patients, namely focusing on tumor-specific factors such as stage, histology, tumor sidedness and other prognostic markers. Awareness needs to be raised in clinical oncology work that the effectiveness of treatment is just as high in patients of advanced age or those with comorbidities, and thus extensive management of these patient groups is mandatory.

Several potential limitations should be considered when interpreting the results of our study. Foremost, it is an explorative study, which implies the risk of selection and information bias. This is especially important considering CCI and aaCCI, both of which were obtained in a retrospective manner. However, the frequency of CCI is very consistent with the literature, thus limiting the risk of implausibility. Furthermore, granting an equal distribution of the high number of prognostic factors (e.g., location of primary, number of metastatic sites, synchronous/metachronous, etc.) known for colorectal cancer is difficult in this setting. We further do not have sufficient data of treatment details such as dose of substances or intervals between the treatments and therefore cannot report on these. The parameters reported were obtained prospectively and monitored through cancer registries. However, as these are real-world data outside a clinical protocol, disease dynamics like disease progression cannot be assessed reliably. We chose OS as a reliable endpoint in order to reduce such biases. However, the strengths of our study is that such information is scare in the literature and of high clinical relevance to provide the translation of progress in oncology into daily practice.

## 5. Conclusions

In our findings, effectiveness of oncological treatment in mCRC appears to be independent of aaCCI or age. Differences found in treatment outcomes are marginal and are likely due to less intense treatment of comorbid or elderly patients. We therefore consider that balancing risk and benefit for treatment decisions should take possible under-treatment of comorbid and older patients into account and recommend the management of comorbidities accompanying the underlying malignant disease.

## Figures and Tables

**Table 1:** Patient demographics.

Variable	Category	*N (%)*
Patients (*n*)	number	1105
Age (Median (IQR))	years	69.00 (60.00, 76.00)
Sex	female	413 (37.4)
	male	692 (62.6)
CCI	0	357 (63.0)
	1 to 2	178 (31.4)
	>2	32 (5.6)
aaCCI	0 to 1	123 (21.7)
	2 to 4	261 (46.0)
	>4	183 (32.3)
Type of treatment	sytemic treatment only	590 (53.4)
	resection of metastases +/− systemic treatment	270 (24.4)
Treated or not treated	treated (resection and/or systemic therapy)	860 (77.8)
	no treatment (BSC)	245 (22.2)
First line treatment	no	342 (31.0)
	yes	763 (69.0)
Second line treatment	no	613 (55.5)
	yes	492 (44.5)
Third line treatment	no	819 (74.1)
	yes	286 (25.9)
Location of primary tumor	right colon	195 (18.1)
	left colon	499 (46.2)
	rectum	385 (35.7)
Time to metastasis	synchronous	771 (69.8)
	metachronous	334 (30.2)
BRAF status	BRAF-mut	41 (3.7)
	BRAF-wt	365 (33.0)
	not analyzed	699 (63.3)
RAS status	RAS-mut	453 (41.0)
	RAS-wt	426 (38.6)
	not analyzed	226 (20.5)
Organs with metastases	>1 organ with metastases	322 (29.2)
	single organ metastasis	781 (70.8)

Values are given in *n* (%) if not otherwise stated; CCI: Charlson Comorbidity Index; aa: age adjusted.

**Table 2:** Clinical outcome in all patients and subgroups defined by comorbidities and age depending on treatment.

Patient Groups	Treatment Groups	HR	CI 90%	OS (Months)	CI 95%
All patients (*n* = 1105)	BSC	1		5.58	(3.88–7.17)
	incl. metastasis resection	0.16	(0.13–0.20)	54.3	(45.96–65.80)
	systemic therapy only	0.45	(0.38–0.52)	19.74	(17.67–22.04)
aaCCI ≤ 3 (*n* = 370)	BSC	1		5.58	(2.73–9.4)
	incl. metastasis resection	0.12	(0.08–0.17)	56.54	(45.96–100.5)
	systemic therapy only	0.3	(0.22–0.39)	26.02	(22.01–29.9)
aaCCI > 3 (*n* = 197)	BSC	1		3.93	(1.97–6.87)
	incl. metastasis resection	0.16	(0.10–0.24)	36.7	(19.81–96.06)
	systemic therapy only	0.39	(0.29–0.51)	17.44	(14.29–21.22)
Age ≤ 70 years (*n* = 609)	BSC	1		5.03	(2.66–9.4)
	incl. metastasis resection	0.12	(0.09–0.15)	58.64	(53.02–78.0)
	systemic therapy only	0.36	(0.28–0.45)	22.77	(20.47–24.9)
Age > 70 years (*n* = 496)	BSC	1		5.91	(3.88–7.33)
	incl. metastasis resection	0.23	(0.18–0.30)	38.04	(30.91–54.30)
	systemic therapy only	0.56	(0.47–0.66)	15.8	(13.70–17.84)

BSC Best supportive care; HR Harzard Ratio; CI Confidence Intervall; CCI Charlson Comorbidity Index; aaCCI age adjusted CCI.

**Table 3:** Treatment intensity and biological features according to comorbidities and age.

**Variable**	**Category**	**aaCCI**		***p* (Exact) **
		**aaCCI ≤ 3**	**aaCCI > 3**	
		***n*** **= 370**	***n*** **= 197**	
Type and intensity of treatment	Receiving best supportive care	46 (12.4)	74 (37.6)	<0.001
	Metastasis resection only	27 (7.3)	17 (8.6)	0.622
	Metastasis resection and systemic treatment	92 (24.9)	19 (9.6)	<0.001
	Receiving systemic treatment only	205 (55.4)	87 (44.2)	0.013
	Receiving first line therapy	297 (80.3)	106 (53.8)	<0.001
	Receiving second line therapy	189 (51.1)	50 (25.4)	<0.001
	Receiving third line or more therapy	91 (24.6)	27 (13.7)	0.002
	1 treatment line only	108 (29.2)	57 (28.9)	1.000
	2 treatment lines only	98 (26.5)	23 (11.7)	<0.001
	3 or more treatment lines	91 (24.6)	26 (13.2)	0.001
Location of primary tumor	Right sided colon cancer	53 (14.6)	39 (20.2)	0.010
	Left sided colon cancer	160 (44.0)	98 (50.8)	
	Rectum cancer	151 (41.5)	56 (29.0)	
Prognostic features	RAS analyzed	325 (87.8)	129 (65.5)	<0.001
	Synchronous disease	266 (71.9)	141 (71.6)	1.000
	>1 organ with metastases	136 (36.8)	59 (30.1)	0.115
		**Age [Years]**		***p* (Exact) **
		**≤70**	**>70**	
		***n*** **= 609**	***n*** **= 496**	
Type and intensity of treatment	Receiving best supportive care	68 (11.2)	177 (35.7)	<0.001
	Metastasis resection only	58 (9.5)	39 (7.9)	0.339
	Metastasis resection and systemic treatment	128 (21.0)	45 (9.1)	<0.001
	Receiving systemic treatment only	355 (58.3)	235 (47.4)	<0.001
	Receiving first line therapy	483 (79.3)	280 (56.5)	<0.001
	Receiving second line therapy	344 (56.5)	148 (29.8)	<0.001
	Receiving third line or more therapy	204 (33.5)	82 (16.5)	<0.001
	1 treatment line only	142 (23.3)	132 (26.6)	0.208
	2 treatment lines only	140 (23.0)	66 (13.3)	<0.001
	3 or more treatment lines	201 (33.0)	82 (16.5)	<0.001
Location of primary tumor	Right sided colon cancer	87 (14.6)	108 (22.3)	<0.001
	Left sided colon cancer	270 (45.5)	229 (47.2)	
	Rectum cancer	237 (39.9)	148 (30.5)	
Prognostic features	RAS analyzed	521 (85.6)	358 (72.2)	<0.001
	Synchronous disease	425 (69.8)	346 (69.8)	1.000
	>1 organ with metastases	190 (31.2)	132 (26.7)	0.097

Numbers are given as *n* (%); aaCCI: age adjusted Charlson Comorbidity Index; *p* (exact) significance level by exact-test.

## Data Availability

The datasets used and/or analyzed during the current study are available from the corresponding author upon request.

## Appendix A

**Table A1:** Cox Regressions including interaction terms age and treatment modalities and aaCCI and treatment modalities, respectively.

**Effects for Age**	**HR**	**z**	**CI 95%**	***p***
Age > 70 years	0.91	−0.62	(0.68–1.22)	0.53
Effect of resection of metastases	0.13	−12.39	(0.09–0.18)	0.00
Effect of systemic treatment only	0.36	−7.25	(0.27–0.47)	0.00
Resection of metastases in age > 70 years	1.68	2.32	(1.08–2.61)	0.02
Sysystemic treatment only in age > 70	1.58	2.57	(1.11–2.23)	0.01
**Effects for aaCCI**				
aaCCI 4–9	1.16	0.74	(0.79–1.70)	0.46
Effect of resection of metastases	0.11	−10.48	(0.07–0.17)	0.00
Effect of systemic treatment only	0.28	−7.19	(0.20–0.39)	0.00
Resection of metastases in aaCCI 4–9	1.22	0.64	(0.81–2.08)	0.52
Sysystemic treatment only in aaCCI 4–9	1.29	1.07	(0.68–1.22)	0.29

HR—Hazard Ratio; z test statistic, CI—Confidence interval; *p*—*p*-value.
